# The Effects of Trends in Osteoporosis Treatment on the Incidence of Fractures

**DOI:** 10.1155/2021/5517247

**Published:** 2021-05-27

**Authors:** Akira Horikawa, Naohisa Miyakoshi, Michio Hongo, Yuji Kasukawa, Yoichi Shimada, Hiroyuki Kodama, Akihisa Sano

**Affiliations:** ^1^Shizuoka Tokusyukai Hospital, 1-11 Surugaku-Simokawahara-Minami, Shizuoka 421-0117, Japan; ^2^Department of Orthopedic Surgery, Akita University Graduate School of Medicine, 1-1-1 Hondo, Akita 010-8543, Japan; ^3^South Akita Orthopedic Clinic, Seiwakai, 96-2 Kaidousita, Syowa-Ookubo, Katagami 018-1401, Japan

## Abstract

**Objective:**

This study focused on the trends in antiosteoporosis drug preferences and compared the incidence of fractures between patients treated orally and those who were exposed to an awareness campaign and assigned to intravenous/subcutaneous treatment.

**Methods:**

Our hospital registry included 1,716 osteoporotic women who were over 65 years of age without preexisting vertebral and nonvertebral fractures over 1 year before this study, with bone mineral density (BMD) < −2.5 standard deviation (SD) and fracture assessment tool (FRAX) score > 20%, who were given 1,337 oral and 379 intravenous/subcutaneous prescriptions to treat their osteoporosis. Self-administered surveys (2012, 2013, 2014, 2015, and 2016) collected data on trends of preferences among nine drugs and fracture prevention using relative risk reduction (RRR).

**Results:**

The number of patients taking oral prescriptions decreased gradually from 2012 to 2016, while the number of patients treated with intravenous and subcutaneous injections increased. The incidence of fracture was lower in patients receiving intravenous and subcutaneous injections than in patients taking oral medications.

**Conclusion:**

These findings indicate a decrease in oral prescriptions for osteoporosis treatment and that treatment for osteoporosis using intravenous or subcutaneous injections of antiosteoporosis drugs is more effective for preventing fractures.

## 1. Introduction

Osteoporosis has been recognized as a major disease of older adults, especially women who often suffer from fractures due to fragility [1]. Recently, many kinds of antiosteoporosis drugs have been developed to improve bone fragility through antibone resorptive (e.g., bisphosphonate) or bone anabolic effects (e.g., teriparatide), and bisphosphonates appear to be used as the first choice for treating osteoporosis, including the treatment of vertebral fractures, in Japanese Centers. Despite the recognized efficacy of bisphosphonates, teriparatide, and other antiosteoporosis drugs with respect to BMD, there are few reports of the preferences of osteoporotic patients [2–4] and comparisons of fracture prevention among these drugs, [5–12] especially in Japan. This study focused on the trends in preferences for antiosteoporosis drugs and compared the incidence of fractures between patients treated orally and those treated with intravenous/subcutaneous drugs.

## 2. Methods

### 2.1. Study Design

A retrospective cohort of patients treated as outpatients for osteoporosis and fractures between 2012 and 2016 at Igarashi Memorial Hospital was examined. Patients for the cohort were selected based on the standard for the diagnosis of osteoporosis: both T score of BMD < −2.5 SD and FRAX in MOF > 20%. DXA was performed by using the DTX-200, Datex DSM, Courtaboeuf Cedex, France. A total of 1716 patients were provided awareness program that, based on their preferences from either oral prescription or intravenous/subcutaneous administration after the usage of these several kinds of antiosteoporotic drugs, including their risks, benefits, and costs associated with treatments which were paid by insurance, was explained. Patients were excluded if they were cognitively impaired, had other illness, had previous treatment of osteoporosis, or had a previous fracture within 1 year. The antiosteoporosis drugs prescribed in this trial were alendronate (oral: *n* = 673, intravenous: *n* = 42), risedronate (oral: *n* = 128), minodronate (oral: *n* = 465), ibandronate (intravenous: *n* = 129), selective estrogen modulator (oral: *n* = 71), teriparatide (subcutaneous, daily: *n* = 118, weekly: *n* = 57), and denosumab (subcutaneous: *n* = 33). The patients were divided into two groups according to the route of drug administration: an oral group and intravenous or subcutaneous (IV/SC) group. Moreover, the IV/SC group was classified into two groups: IV bisphosphonate (alendronate and ibandronate) and the anabolic group (teriparatide and denosumab). All eligible patients were over 65 years of age, and the average of the follow-up period for this trial was around 2 years. For the surveillance study, subjects were asked to identify fractures they had experienced during this treatment for any of the specified locations: the clavicle, upper arm, wrist, spine, rib, hip, pelvis, upper leg, and lower leg. This study was conducted according to the Helsinki Declaration, and the medical ethics committee of Akita University Graduate School of Medicine approved this study (approval number: 1970).

### 2.2. Data Extraction and Study Selection

Information, including patient numbers, sample size, past history of medication, clinical setting, and duration of the follow-up period, was extracted. Dichotomous data were used for reporting the number of patients in each treatment group, adherence to treatment, and the fracture rate. For fracture evaluation, magnetic resonance imaging and the Genant method, especially for vertebral fractures, were used. The fracture rate was the incidence of new nonvertebral fractures based on radiological findings [13]. The fracture rate was reported as relative risk reduction (RRR) with a 95% confidence interval (CI) for direct comparisons.

### 2.3. Data Synthesis and Analysis

Data for age, BMD of the forearm, FRAX, previous history of treatment for osteoporosis, adherence to treatment, and fracture rate were evaluated. Information on demographic characteristics of the subjects, osteoporotic condition, and outcomes of fractures was extracted from each clinical report and tabulated in Microsoft Excel spreadsheets. For each of the dependent variables, several explanatory variables including duration of treatment, number of patients, age, sex, age, body mass index (BMI), and previous history of medications were examined. Statistical analysis was performed using Microsoft Office Excel and the Statcel 3 program (OMS, Inc., Hyogo, Japan). Each subject was analyzed by the unpaired *t-*test to compare differences between the two groups, except for the incidence of fractures. The chi-squared test was used to evaluate the significance of differences in FRAX. All results of statistical tests were regarded as significant with *p* < 0.05. The incidence of fracture was calculated by the RRR with a 2-way contingency table analysis (Center for Evidence-Based Medicine: http://www.grade-jpn.com/2x2.html). The RRR was based on the comparison with oral alendronate, which was the most prescribed drug (*n* = 672).

## 3. Results

### 3.1. Number of Participants

**Figure 1:**
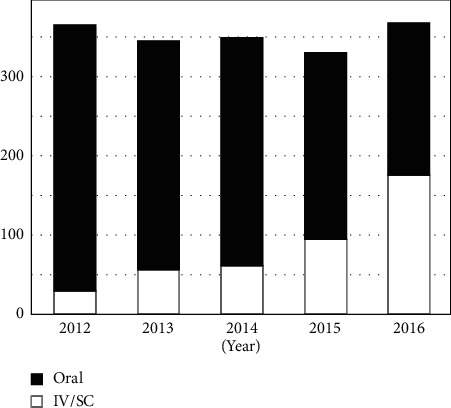
Chronological changes in the number of osteoporosis patients treated by oral or intravenous/subcutaneous drugs.

The accumulated number of patients was 1337 on oral prescriptions and 379 in the IV/SC group. The IV/SC group had significantly older age, higher FRAX score, and a significantly lower incidence of fracture than the oral group. There were no significant differences between the groups in BMI, BMD, and past history of medications (Table 1). The number of patients taking oral prescriptions decreased gradually, and the number of patients receiving intravenous or subcutaneous drugs increased with time (Figure 1). In the oral group, alendronate users were markedly decreasing, but risedronate and SERM users increased gradually. In the IV/SC group, there was a dramatic increase in the number of ibandronate users, whereas other groups showed little change, except for denosumab and weekly teriparatide (Table 2).

The IV/SC group showed a higher rate of adherence to treatment than the oral group (Table 3).

### 3.2. Change of Bone Turnover Markers

**Figure 2:**
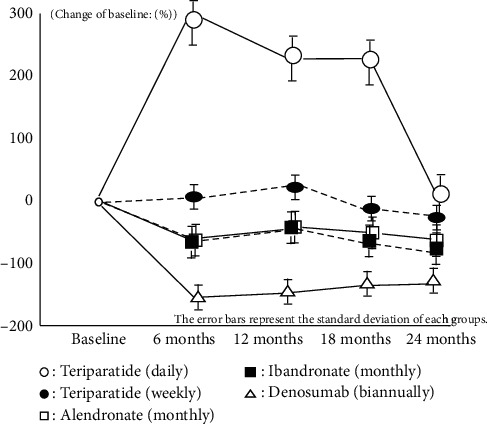
Change of TRACP-5b or NTx.

**Figure 3:**
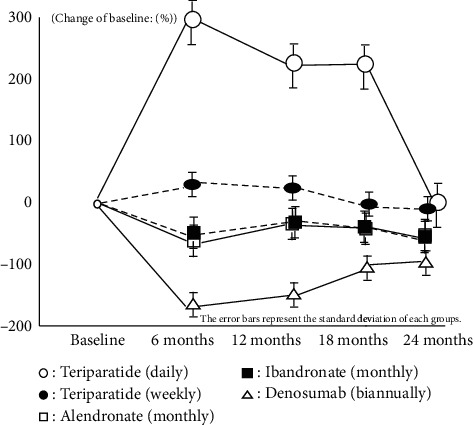
Change of P1NP or BAP.

**Figure 4:**
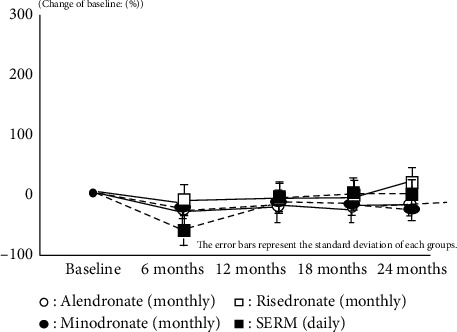
Change of NTx.

**Figure 5:**
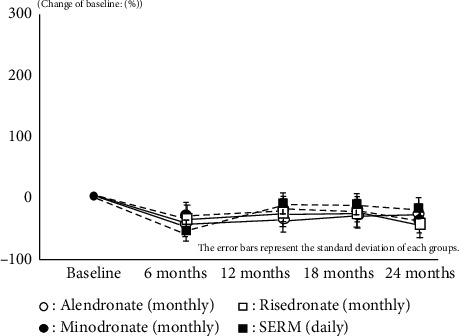
Change of BAP.

Both bone resorption and bone formation markers also showed almost same results which have already been reported by Japanese clinical trial phase 3 in every IV/SC and oral groups. These results imply that all medications were effective during this treatment period on the basis of restriction of bone turnover marker and anabolic window (Figures 2–5).

### 3.3. Adverse Events

The number of incidence of adverse events in each drug is shown in Table 4. The ratio of frequency of them was similar as it has already been reported in a phase 3 trial. Weekly teriparatide has a tendency for high ratio.

Incidence of fracture (number of new vertebral or nonvertebral fractures).

The oral group had a significantly higher incidence of fracture than the IV/SC group, which had a high RRR, and the anabolic group appeared to prevent fractures the most in patients with osteoporosis (Table 5).

A comparison of the fracture rate between the IV/SC group and the major oral drug (alendronate, *n* = 672) showed that teriparatide and denosumab had the strongest fracture prevention effect (Table 6).

## 4. Discussion

The number of oral medications has been decreasing in tandem with the increase in the number of intravenous and subcutaneous medications for osteoporosis in the USA [2], like the phenomenon in our hospital. This phenomenon is similar to that in other countries such as France and Germany with low adherence rates to antiosteoporosis treatment [14, 15], and it may also stem from the drug holidays of bisphosphonates [16]. Barcenilla-Wong [3] implied that cost performance and attention to the treatment of osteoporosis among the patient's characteristics were related to this background, and Hilligsman et al. [4] mentioned that osteoporotic patients prefer monthly oral prescriptions or biannual injections, which means an interval protocol. As is the case in our hospital, the trend reflects Wysconski's and Greene report that osteoporotic patients switched from oral to intravenous, although there might be some bias due to the awareness program [2]. In addition, the present patients maintained high adherence to treatment, especially in the IV/SC group, which may have been due to the awareness program. Paramedical staff who were mainly nurse may have contributed to these results by giving guidance on how to use the drugs repeatedly. To provide different routes of treatment and better adherence rate, we should focus on the establishment of osteoporosis liaison service, that is, the system of integration of many paramedical staff such as nurse, radiologic technologist, physical therapist, public health nurse, and many assistants, and try to prevent osteoporotic fragile fracture by linking and sharing their information to each other.

Next, the trends in prevention of osteoporotic fracture were investigated for the several kinds of therapies. The data analysis shown below found that, in the IV/SC group, especially the greatest preventive effect was seen with teriparatide, followed by denosumab, ibandronate, and alendronate in order.

Although there were a few reports comparing BMD and fracture rates among several kinds of drugs in the treatment of osteoporosis [5–12], our awareness campaign showed about a 50% reduction of fracture in the IV/SC group compared to the oral group (RRR = 55%). In regard to this point, Carolyn [9] suggested that bisphosphonates, denosumab, and teriparatide decrease fractures compared with placebo despite the lack of data comparing their efficacies. Moreover, Migliore suggested that the drugs with the greatest effects for preventing osteoporotic fractures are denosumab, ibandronate, alendronate, and risedronate in that order in a mixed treatment comparison meta-analysis [12]. These results are in agreement with the present results based on the RRRs. On the other hand, Nakamura et al. [6] suggested that intravenous ibandronate treatment was more effective for the prevention of fractures in osteoporotic patients than oral risedronate treatment. In addition, Zhang et al. [7] and Hopkins et al. [8] reported that teriparatide and denosumab decreased vertebral fractures more than bisphosphonates. Considering these reports and the present results, intravenous or subcutaneous treatment of osteoporosis might prevent fractures better than oral treatment. Teriparatide and denosumab, in particular, have stronger fracture prevention effects than bisphosphonates. Moreover, when looking at the prevention of fractures by location, vertebral and nonvertebral fractures were strongly reduced by intravenous and subcutaneous injection therapies. These results support our awareness campaign about osteoporosis treatment, and therefore, intravenous and subcutaneous treatment is recommended from the perspective of adherence and the effect on fracture prevention [17–25].

Although this study has multiple methodological limitations including heterogeneity in the comparing groups and types of awareness program, the former drug used anabolic vs. antiresorptive that compared between new off-labeled drug treatment and the present mainstream treatment of osteoporosis. That implies for the testimony of effectiveness whether new anabolic drug treatment is more effective against the present antiresorptive drug treatment or not. The latter, although this awareness program was based on the patient's preferences, may be focused on the injectable groups rather than oral antiresorptive groups due to the patient's family assistance. Therefore, the distribution of these treatments would lead to being inadequate and heterogenous.

Finally, we have some bias, including the number, duration, and patient's choice of medication because zoledronic acid and romosozumab, which have strong fracture prevention effects, could not be used because they were introduced to the Japanese market recently [26–32]. Further investigations including trials of these medications will be needed in the near future.

In summary, these results showed a decrease in the number of oral prescription drugs for osteoporosis treatment, and intravenous and subcutaneous treatment for osteoporosis was more effective in fracture prevention.

## 5. Conclusions

The trends in treatment for osteoporosis were examined, and the fracture rates were compared among nine osteoporosis drugs. The results showed that the number of patients given oral prescription decreased gradually from 2012 to 2016, while the number of intravenous and subcutaneous injections increased, and the fracture rate was relatively low in the intravenous and subcutaneous injection patients compared to the oral prescription patients.

## Figures and Tables

**Table 1:** Baseline variables by group.

	Oral group	IV/SC group	*p* value
Number	1487(150)	457(78)	
Age (y)	79.3 ± 7.9	81.2 ± 6.4	0.200^a^
BMI (kg/m^2^)	25 ± 4.0	24.1 ± 3.6	0.201^a^
BMD: femur (g/cm^2^)	0.464 ± 0.035	0.491 ± 0.052	0.538^a^
BMD: lumbar spine (g/cm^2^)	0.585 ± 0.042	0.552 ± 0.028	0.052^a^
FRAX	20.6	25.9	0.158^b^
Previous history of treatment	150	78	0.052
Alendronate	20	11	
Risedronate	30	13	
Minodronate	18	8	
SERM	46	22	
Vit D	32	17	
Ca supplement	4	7	

^a^Student's *t*-test, ^b^*χ*^2^ test, ( ): number of previous history of treatment.

**Table 2:** Chronological changes in the numbers for osteoporosis treatment: oral prescriptions and intravenous or subcutaneous prescriptions.

	Alendronate	Risedronate	Minodronate	SERM	
2012	208	19	100	8	
2013	145	21	105	14	
2014	140	25	105	17	
2015	107	33	80	16	
2016	72	30	75	16	

	Teriparatide (daily)	Teriparatide (weekly)	Alendronate (monthly)	Ibandronate (monthly)	Denosumab (biannually)
2012	22	7	1	0	0
2013	27	5	12	13	0
2014	22	10	11	18	1
2015	31	16	10	31	7
2016	26	24	10	88	28

Adherence to treatment (percentage staying on medication without discontinuation).

**Table 3:** Comparisons of adherence to treatment (%): Student's *t*-test.

	Oral group	IV/SC group	*p* value
2012	75	100	0.036
2013	80	93	0.044
2014	78	90	0.045
2015	80	88	0.048
2016	83	98	0.041

IV/SC: intravenous/subcutaneous.

**Table 4:** Comparisons of adverse events.

IV/SC group	Number (*n*)	Ratio of incidence (%)	Ratio of incidence in phase 3 (%)
Teriparatide (daily)	12	10	12–13
Teriparatide (weekly)	10	28	30–50
Denosumab (biannually)	4	11	12
Ibandronate (monthly)	13	9	10
Alendronate (monthly)	2	5	7

Oral group	Number (*n*)	Ratio of incidence (%)	Ratio of incidence in phase 3 (%)
Alendronate (monthly)	45	6.7	10
Minodronate (monthly)	5	1.1	Unknown
SERM (daily)	1	1.4	Under 1
Risedronate (monthly)	2	1.6	1–5

Phase 3: Japanese clinical trial final stage before approval; IV/SC: intravenous/subcutaneous.

**Table 5:** Comparisons of the incidence of fracture: oral vs. IV/SC group, oral vs. IV/Bis group, and oral vs. anabolic group.

	Oral group	IV/SC group	RRR	CI
Fracture number	32 (2.4%)	4 (1.1%)	0.559	−0.183–0.837
(Total number)	(1337)	(379)		

	Oral group	IV/bis group	RRR	CI
Fracture number	32 (2.4%)	4 (2.3%)	0.021	−1.264–0.615
(Total number)	(1337)	(171)		

	Oral group	Anabolic group	RRR	CI
Fracture number	32 (2.4%)	0 (0%)	1.000	0.219–1.000
(Total number)	(1337)	(208)		

IV/SC: intravenous/subcutaneous, RRR: relative risk reduction, CI: confidence interval IV/bis: intravenous bisphosphonate, RRR: relative risk reduction, CI: confidence interval IV/SC: intravenous/subcutaneous, RRR: relative risk reduction, CI: confidence interval.

**Table 6:** Comparisons of the incidence of fracture (intravenous and subcutaneous).

	Number	RRR	CI
Teriparatide (daily, *n* = 128)	0 (0%)	1.000	−0.828–1.000
Teriparatide (weekly, *n* = 62)	0 (0%)	1.000	−1.994–1.000
Denosumab (biannually, *n* = 36)	0 (0%)	1.000	−4.294–1.000
Ibandronate (monthly, *n* = 150)	2 (1.333%)	−0.031	−2.469–0.697
Alendronate (monthly, *n* = 44)	2 (4.545%)	−1.398	−8.405–0.413

## Data Availability

The data used to support the findings of this study are restricted by the medical ethics committee of Akita University Graduate School of Medicine. Data are available from Naohisa Miyakoshi (miyakosh@doc.med.akita-u.ac.jp) for researchers who meet the criteria for access to confidential data.

## References

[1] World Health organization (2004). *WHO Scientific Group on the Assessment of Osteoporosis at Primary Health Care Level”*.

[2] Wysowski D. K., Greene P. (2013). Trends in osteoporosis treatment with oral and intravenous bisphosphonates in the United States, 2002–2012. *Bone*.

[3] Barcenilla-Wong A. L., Chen J. S., March L. M. (2014). Concern and risk perception: effects on osteoprotective behaviour. *Journal of Osteoporosis*.

[4] Hiligsmann M., Dellaert B. G., Dirksen C. D. (2014). Patients’ preferences for osteoporosis drug treatment: a discrete-choice experiment. *Arthritis Research & Therapy*.

[5] Chen L. X., Zhou Z. R., Li Y. L. (2015). Comparison of bone mineral density in lumbar spine and fracture rate among eight drugs in treatment of osteoporosis in men: a network meta-analusis. *PLoS One*.

[6] Nakamura T., Nakano T., Nakano T. (2013). Clinical efficacy on fracture risk and safety of 0.5 mg or 1 mg/month intravenous ibandronate versus 2.5 mg/day oral risedronate in patients with primary osteoporosis. *Calcified Tissue International*.

[7] Zhang L., Pang Y., Shi Y. (2015). Indirect comparison of teriparatide, denosumab, and oral bisphosphonates for the prevention of vertebral and nonvertebral fractures in postmenopausal women with osteoporosis. *Menopause*.

[8] Hopkins R. B., Goeree R., Pullenayegum E. (2011). The relative efficacy of nine osteoporosis medications for reducing the rate of fractures in post-menopausal women. *BMC Musculoskeletal Disorders*.

[9] Carolyn J. C., Syde J. N., Allison D. M. (2014). Comparative effectiveness of pharmacologic treatments to prevent fractures. *Annals of Internal Medicine*.

[10] Rizzoli R. (2011). Bisphosphonates for post-menopausal osteoporosis: are they all the same?. *QJM: An International Journal of Medicine*.

[11] Zhou J., Wang T., Zhao X., Miller D. R., Zhai S. (2016). Comparative efficacy of bisphosphonates to prevent fracture in men with osteoporosis: a systematic review with network meta-analyses. *Rheumatology and Therapy*.

[12] Migliore A., Broccoli S., Massafra U., Cassol M., Frediani B. (2013). Ranking antireabsorptive agents to prevent vertebral fractures in postmenopausal osteoporosis by mixed treatment comparison meta-analysis. *European Review for Medical and Pharmacological Sciences*.

[13] Genant H. K., Jergas M., Palermo L (1996). Comparison of semiquantitative visual and quantitative morphometric assessment of prevalent and incident vertebral fractures in osteoporosis. *Journal of Bone and Mineral Research*.

[14] Kanis J. A. (1994). Assessment of fracture risk and its application to screening for postmenopausal osteoporosis: synopsis of a WHO report. WHO study group. *Osteoporosis International*.

[15] Belhassen M., Confavreux C. B., Cortet B., Lamezec L., Ginoux M., Van Ganse E. (2017). Anti-osteoporotic treatments in France: initiation, persistence and switches over 6 years of follow-up. *Osteoporosis International*.

[16] Adler R. A., Fuleihan G. E., Bauer D. C. (2015). Managing osteoprosis in patients on long-term bisphosphonate treatment: report of a task force of the American society for bone and mineral research. *Journal of Bone and Mineral Research*.

[17] Hadji P., Claus V., Ziller V., Intorcia M., Kostev K., Steinle T. (2012). GRAND: the German retrospective cohort analysis on compliance and persistence and the associated risk of fractures in osteoporotic women treated with oral bisphosphonates. *Osteoporosis International*.

[18] Nakamura T., Fukunaga M., Nakano T. (2017). Efficacy and safety of once-yearly zoledronic acid in Japanese patients with primary osteoporosis: two-year results from a randomized placebo-controlled double-blind study (ZOledroNate treatment in efficacy to osteoporosis; ZONE study). *Osteoporosis International*.

[19] Neer R. M., Arnaud C. D., Zanchetta J. R. (2001). Effect of parathyroid hormone (1–34) on fractures and bone mineral density in postmenopausal women with osteoporosis. *New England Journal of Medicine*.

[20] Nakamura T., Sugimoto T., Nakano T. (2012). Randomized teriparatide [human parathyroid hormone (PTH) 1–34] once-weekly efficacy research (TOWER) trial for examining the reduction in new vertebral fractures in subjects with primary osteoporosis and high fracture risk. *The Journal of Clinical Endocrinology & Metabolism*.

[21] Nakamura T., Matsumoto T., Sugimoto T. (2014). Clinical trials express:fracture risk reduction with denosumab in Japanese postmenopausal women and men with osteoporosis: denosumab fracture intervention randomized placebo controlled trial (DIRECT). *The Journal of Clinical Endocrinology & Metabolism*.

[22] Cummings S. R., Martin J. S., McClung M. R. (2009). Denosumab for prevention of fractures in postmenopausal women with osteoporosis. *New England Journal of Medicine*.

[23] Shiro T., Tatsuhiko K., Toshitsugu S., Toshitaka N., Masataka S. (2014). Changes in bone mineral density, bone turnover markers, and vertebral fracture risk reduction with once weekly teriparatide. *Current Medical Research and Opinion*.

[24] Chesnut C. H., Skag A., Christiansen C. (2004). Effects of oral ibandronate administered daily or intermittently on fracture risk in postmenopausal osteoporosis. *Journal of Bone and Mineral Research*.

[25] Wells G. A., Cranney A., Zytaruk N. (2007). Alendronate for the primary and secondary prevention of osteoporotic fractures in postmenopausal women. *Cochrane Database of Systematic Reviews*.

[26] Black D. M., Delmas P. D., Eastell R. (2001). Once-yearly zoledronic acid for treatment of postmenopausal osteoporosis. *The New England Journal of Medicine*.

[27] Kenneth W. L., Cathleen S. C., Jay S. M. (2007). For the HORIZON recurent fracture trial. *The New England Journal of Medicine*.

[28] Eric S. O., Paul D. M., Jonathan D. A. (2010). Efficacy and safety of a once-yearly i.v. infusion of zoledronic acid 5 mg versus a once-weekly 70 mg oral alendronate in the treatment of male osteoporosis: a randomized , multicenter, double-blind active-controlled study. *Journal of Bone and Mineral Research*.

[29] Sohita D. (2016). Zoledronic acid (reclast®, aclasta®): a review in osteoporosis. *Drugs*.

[30] Black D. M., Reid I. R., Boonen S. (2012). The effect of 3 versus 6 years of zoledronic acid treatment of osteoporosis: a randomized extension to the HORIZON‐pivotal fracture trial (PFT). *Journal of Bone and Mineral Research*.

[31] Hwang J.-S., Chin L.-S., Chen J.-F. (2011). The effects of intravenous zoledronic acid in Chinese women with postmenopausal osteoporosis. *Journal of Bone and Mineral Metabolism*.

[32] Saag K. G., Petersen J., Brandi M. L. (2017). Romosozumab or alendronate for fracture prevention in women with osteoporosis. *The New England Journal of Medicine*.

